# Children and Adults Both Learn Motor Sequences Quickly, But Do So Differently

**DOI:** 10.3389/fpsyg.2017.00158

**Published:** 2017-02-07

**Authors:** Yue Du, Nadia C. Valentini, Min J. Kim, Jill Whitall, Jane E. Clark

**Affiliations:** ^1^Department of Kinesiology, University of Maryland, College Park, College ParkMD, USA; ^2^Department of Physical Education, Physical Therapy and Dance, Federal University of Rio Grande do SulPorto Alegre, Brazil; ^3^Department of Mechanical Engineering, College of Engineering, Kyung Hee UniversitySuwon, South Korea; ^4^Department of Physical Education, Seoul National UniversitySeoul, South Korea; ^5^Department of Physical Therapy and Rehabilitation Science, School of Medicine, University of Maryland, BaltimoreBaltimore, MD, USA; ^6^Faculty of Health Sciences, University of SouthamptonSouthampton, UK; ^7^Neuroscience and Cognitive Science Program, University of Maryland, College ParkCollege Park, MD, USA

**Keywords:** fast sequence learning, age-related, online process, oﬄine process, implicit sequence learning, declarative sequence knowledge, fatigue, task pacing

## Abstract

Both children and adults can learn motor sequences quickly in one learning session, yet little is known about potential age-related processes that underlie this fast sequence acquisition. Here, we examined the progressive performance changes in a one-session modified serial reaction time task in 6- and 10-year-old children and adults. We found that rapid sequence learning, as reflected by reaction time (RT), was comparable between groups. The learning was expressed through two behavioral processes: online progressive changes in RT while the task was performed in a continuous manner and oﬄine changes in RT that emerged following a short rest. These oﬄine and online RT changes were age-related; learning in 6-year-olds was primarily reflected through the oﬄine process. In contrast, learning in adults was reflected through the online process; and both online and oﬄine processes occurred concurrently in 10-year-olds. Our results suggest that early rapid sequence learning has a developmental profile. Although the unifying mechanism underlying these two age-related processes is unclear, we discuss possible explanations that need to be systematically elucidated in future studies.

## Introduction

Throughout our day, we effortlessly produce sequences of actions from getting out of bed in the morning, tying our shoes, to pouring a cup of coffee and drinking it. While these motor sequences comprise much of what we do in our activities of daily living, their acquisition is not altogether well understood. For example, motor sequence acquisition in adults (Willingham et al., 1989; Stadler and Frensch, 1998) and children (Meulemans et al., 1998; Thomas and Nelson, 2001; Weiermann and Meier, 2012) have been studied using the serial reaction time (SRT) task (Nissen and Bullemer, 1987). The SRT task usually consists of a single learning session of 4–8 learning blocks with a short break between blocks. In each block, participants respond as fast as possible to a sequence of visual stimuli. The visual stimuli are presented in a fixed order that is unknown to participants. The mean reaction time (RT) of each block decreases as participants repeatedly practice the same sequence, while the mean RT becomes slower when a novel sequence that has not been practiced is introduced (Nissen and Bullemer, 1987; Willingham et al., 1989). As revealed by the mean RT improvement to the same sequence and a reversal of improvement to a new sequence, learning of sequences in adults and children as young as 6 years of age develop quickly (over a single learning session) and to a comparable level (Meulemans et al., 1998). Little is known, however, about the age-related processes underlying this sequence acquisition that takes place so quickly over the course of one learning session. The purpose of this study, therefore, was to examine whether the same or different processes underlie motor sequence learning in children and adults. That is, in addition to the mean RT that indicates the learning of sequences, we aimed to examine the trial-by-trial RT changes within and between blocks when adults and children perform the SRT task.

In our recent study where adults learned a fixed repeating sequence (Du et al., 2016), we observed a trial-by-trial RT decrease within each learning block when adults continuously responded to visual stimuli (i.e., online process), while RT remained the same after a short rest between blocks (with no physical practice; i.e., oﬄine process). Thus, learning of a fixed sequence in adults was primarily expressed by the trial-by-trial online RT improvement and not oﬄine RT improvement. Upon completing the SRT task, adults acquired declarative (explicit) knowledge of the fixed sequence, although they were not informed of the sequence before or during learning. When the sequence was manipulated to be more complex, adults learned the sequence as well, but without acquiring declarative sequence knowledge. And importantly, the adults’ sequence acquisition exhibited oﬄine rather than online processes of learning (Du et al., 2016). These results suggest that the strengths of online and oﬄine process may be accompanied by the degree of task implicitness, that is, whether an individual could acquire the declarative knowledge of the learned sequence. To date, no studies have examined whether sequence learning in children is reflected by the online or oﬄine process. Since the online process requires iterative computations (Cleeremans and McClelland, 1991; Bornstein and Daw, 2012, 2013; Verstynen et al., 2012) that involve using the previous trial’s information to update performance on the next trial, it may impose demanding computational requirements for children. Thus, we expected weaker online RT improvements in children compared to adults. Given that children are able to learn motor sequences at a comparable level to adults (Meulemans et al., 1998; Karatekin et al., 2007), a stronger oﬄine process would need to be developed to compensate for a weaker online process and ensure that children have the same capability to learn sequences. Moreover, the fact that children are less able to acquire the declarative knowledge of a learned fixed sequence than adults (Weiermann and Meier, 2012) also leads us to conjecture a weaker online and stronger oﬄine process in children’s learning.

Here, we asked adults and children to perform a foot-stepping version of the SRT task. The SRT task consisted of six learning blocks with a 3 min break between two blocks. In each block, 6- and 10-year-old children and adults responded to a 100-trial sequence of visual stimuli that followed either a fixed order A (sequence A in blocks 1–4 and 6) or a fixed order B (sequence B in block 5). We measured the RT decrease in sequence A between blocks 1 and 4 (Nissen and Bullemer, 1987), as well as the RT increase from blocks 4 to 5 when sequence B was introduced. Since the former change may partially be attributed to general motor improvement (Robertson, 2007), the latter change is considered as a better indicator of sequence learning (Nissen and Bullemer, 1987; Willingham et al., 1989). Consistent with the literature (Meulemans et al., 1998; Karatekin et al., 2007), we expected the same RT changes (from blocks 1 to 4 as well as from blocks 4 to 5) between children and adults. To confirm the previous finding that adults acquire more declarative sequence knowledge than children (Weiermann and Meier, 2012), we measured the declarative knowledge of sequence A that children and adults acquired after the SRT task. Furthermore, we measured online process as the change in RT within each learning block. The change in RT immediately before and after each rest was computed to infer the oﬄine process. We examined these online and oﬄine RT changes in children and adults to determine whether motor sequence learning is reflected by age-related processes, and, particularly, whether sequence learning in adults exhibits an online improvement in RT and that in children is primarily reflected by an oﬄine process.

## Materials and Methods

This study was approved by the Institutional Review Board at the University of Maryland, College Park and it was performed in accordance with the approved guidelines. Signed consent forms from the adult participants/parents and assent forms from child participants were received prior to their participation. Each participant received a $15 monetary compensation upon the completion of the experiment. Child participants also received a small toy prize for completing the Movement Assessment Battery for Children 2 (MABC2) (Henderson et al., 2007).

### Participants

Ten 6-year-old children (6.65 ± 0.83 years, male = 6) and 13 10-year-old children (10.5 ± 0.68 years, male = 5) were recruited for this study. Prior to the experiment, children were screened by the MABC2 to determine if movement difficulties existed. One 10-year-old male child was excluded because he scored below the 5th percentile on the MABC2. The remaining 22 children scored above the 25th percentile and so were included in this study. Ten non-musician adults (20.47 ± 0.9 years, male = 5) were recruited from the University of Maryland, College Park. For one adult participant, data from the last block (i.e., block 6) were excluded from the data analysis owing to unexpected equipment problems when he performed the task. Prior to participation, adults completed a neurological health questionnaire. No participants reported neurological health issues. In addition, participants were screened for their experience with the video game, Dance Dance Revolution (DDR) since the SRT task we employed was similar to the DDR video game. All participants had little DDR experience (i.e., equal or less than 2 h experience) and no participants had played the DDR game more than twice in the past year.

### Experimental Task

**FIGURE 1:**
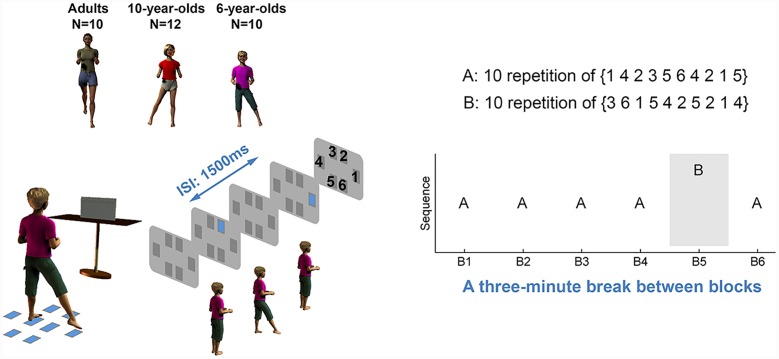
**Experimental setup and protocol**. Ten 6-year-old children, 13 10-year-old children (12 were included for data analyses), and 10 adults performed a foot stepping serial reaction time (SRT) task. Participants responded to each visual stimulus as quickly and accurately as possible by stepping to the spatially matched target on the floor. The stepping performance was measured by reaction time and movement time. In blocks 1–4, the visual stimuli followed sequence A (10 repetitions of 1423564215). In block 5, the stimulus followed sequence B (10 repetitions of 3615425214). Each number was associated with one spatially located square, but the numbers were not displayed to participants. Both sequences consist of 100 trials and each stimulus appeared 1500 ms after the preceding stimulus. After each learning block, participants had a 3-min rest. Participants were not told that the stimuli followed a sequence until they completed all six blocks.

Participants stood on a home position (two 18 cm × 11 cm felt mats) and performed a whack-a-mole type game with sequential foot stepping. Six stepping targets (six 12 cm × 12 cm felt mats) were positioned to the front, back, and side of the home position (see **Figure 1**). The distances between the targets and home position were marked at 60% of the longest step that the participants were able to accomplish in each direction. The step length to each of the six blocks, therefore, was comfortable and less than maximum leg reach. Six visual cues (i.e., six holes) were presented on the monitor positioned in front of the participants. These visual cues were spatially compatible with the targets on the floor. One mole at a time successively popped up from one of the six holes to represent the sequence order (see **Figure 1**). A laptop computer with a customized Labview (National Instruments, Austin, TX, USA) program controlled the sequential stimuli. A Vicon motion capture system (Oxford Metrics, Oxford, UK) recorded the real-time three-dimensional positions of reflective markers attached to the participants’ big toes, heels (calcaneus), and the 5th metatarsal on both feet with a sampling frequency of 200 Hz.

### Procedures

Participants stood on the home position before starting each experimental block. They were instructed to step to the appropriate target on the floor as quickly and accurately as possible when the mole appeared in the corresponding location on the screen. They were required not to step to targets 1, 2, and 6 with the left foot and targets 3, 4, and 5 with the right foot (**Figure 1**). After each step, they were required to step back to the home position for the next stimulus, appearing 1500 ms after the previous stimulus. We chose the inter-stimulus-interval (ISI) of 1500 ms because it was long enough for adults and children to step to the target and return to the home position. One point worthy of emphasizing is that the constant ISI resulted in a relatively shorter response-stimulus-interval in children because of their longer response time (see Results). The shorter response-stimulus-interval in children may impair the online process because the online process requires iterative mental computations that need an adequate amount of time before the next stimulus appears. However, a subsequent study from our lab asked children and adults to perform a self-paced SRT task where the response-stimulus-interval was fixed and thus ISI was dictated by an individual’s own response speed. This subsequent study provided preliminary evidence that the online and oﬄine process in children and adults were unlikely to be influenced by the task pacing condition (Du, 2016). During the whole experiment, an accurate hit on the target mat or home position was encouraged, but not strictly required because, during the continuous stepping movement, participants, and especially children, would shift their positions slightly. However, stepping in the right direction was required. Before the experimental trial blocks began, participants practiced with a random sequence of 36 trials to become familiar with the task.

In the experiment, participants performed six blocks of foot stepping movements. For blocks 1–4, the stimuli followed sequence A (10 repetitions of the sequence 1423564215). Each number was associated with one spatially located square, as shown in **Figure 1**, but the numbers were not displayed to participants nor described to them as such. A novel sequence (sequence B), 10 repetitions of the sequence 3615425214, was provided for block 5 followed by block 6 when participants again performed sequence A. Thus, each block consisted of 100 steps (10 repetitions of 10 steps). After each block, participants took a short break lasting about 3 min. Until the completion of all six blocks, participants were not informed that the visual stimuli followed any order. Upon completion of all six blocks, participants were asked whether they noticed there was a sequence to the presentation of the visual stimuli. Subsequently, participants were given four different 10-element long sequences and were asked to choose the one they thought had appeared in their task (i.e., recognition task I). Participants were then asked to complete a recognition task II that consisted of four trials. In each trial, they were given four sequence segments and were asked to choose the ones they thought had appeared in the task. Finally, participants were asked to recall and write down the sequence. These questions were asked to investigate whether participants had declarative knowledge of the sequence they had been practicing.

### Data Analysis

Reaction time (RT) that measured the temporal difference between the stimulus onset and the movement initiation, as well as movement time (MT) that quantify the time elapsed from the movement initiation to the end of movement, were derived in MATLAB^TM^ (MathWorks, Natick, MA, USA). Specifically, the time series of the three-dimension trajectory of markers on the toes, heels, and the fifth metatarsals were filtered by an eighth-order Butterworth filter with a cutoff frequency of 10 Hz. We marked the movement onset as the first sample when the foot reached 10% of the maximum height of movement. The end point of stepping was defined as the time when the foot dropped to the same height as the onset. Steps were considered an error and discarded if one of the following two conditions occurred: (1) stepping to a wrong target; or, (2) stepping to the correct target but from other targets and not from the home position as required. A trial’s RT or MT also was excluded if its absolute magnitude was out of the range from (μ - 2.5 × δ) to (μ + 2.5 × δ), where μ and δ are the mean and standard deviation of the raw RT or MT for each block (Ratcliff, 1993). We chose this specific range rather than other smaller ones to preserve as much of the raw RT and MT data in our data analysis as possible. These criteria resulted in excluding 1.98 ± 0.42% (mean ± standard error) of the RTs in adults, 2.28 ± 0.29% in 10-year-olds, and 2.75 ± 0.36% of the RTs in 6-year-olds in each block. There were no outliers in terms of MT in all age groups.

We derived several variables to quantify sequence learning and its progressive processes in RT and MT. The block’s mean RT (BMRT) was employed to assess the summative performance throughout the entire task. The magnitude of learning was measured by the BMRT improvement from blocks 1 to 4 as well as the BMRT difference between sequence A in block 4 and the novel sequence B in block 5. Since the performance change from blocks 1 to 4 may be partially attributed to general motor improvement (i.e., they became more familiar with the task), the difference between blocks 4 and 5 was suggested as a better indicator of sequence learning (Nissen and Bullemer, 1987; Willingham et al., 1989; Robertson, 2007). We chose the mean rather than median of RT as: (1) the sample median may provide a biased estimation of RT (Miller, 1988); and (2) after excluding outliers, the mean RT represented the performance as effectively as the median of raw RT, which was revealed by a significantly high correlation (*r* = 0.98, *p* < 0.00001 for all three age groups). The same set of variables was calculated for MT.

To assess oﬄine and online processes, the mean RT for each repetition of 10 steps (RMRT) was used. The magnitude of oﬄine gain was computed as the discrepancy between the last RMRT in one block and the first RMRT in the succeeding block. Online change in RT was defined as the RT change that takes place within block and was computed as the difference between the first and last RMRT in the same block. A positive value of online or oﬄine change indicates increased RT (i.e., RT became slower) while a negative value means decreased RT (i.e., RT became faster). To determine the overall RT changes during oﬄine and online periods when participants were learning the motor sequence through blocks 1 to 4, we averaged oﬄine and online RT changes before block 5 in which a novel sequence B was introduced. We used average rather than total RT change since there were four blocks (i.e., blocks 1 to 4) where online learning could take place and only three breaks (i.e., between blocks 1 and 2, blocks 2 and 3, and blocks 3 and 4) where oﬄine learning could occur before a novel sequence B was given. The same set of variables was calculated for MT.

One confounding factor underlying the oﬄine RT improvement is the emergence of fatigue (Rieth et al., 2010). Fatigue may accumulate with practice and deteriorate performance before a rest. The fatigue effect dissipates following the rest, leading to an artificial oﬄine improvement. To correct for any fatigue effects, we calculated corrected oﬄine processes in which RTs that exhibited an online increase in the previous block were subtracted from the following oﬄine RT change. In other words, it was the difference in the first RMRT between two consecutive blocks. Thus, the correct oﬄine change in RT represented how much RT improved with respect to its level before possible fatigue (i.e., if RT increase online) took place in the previous block. To further examine the likelihood of a fatigue effect, we followed the method in a previous study that computed the mean RT for the first 50 trials (i.e., the first half) and second 50 trials (i.e., the second half) in each block to examine whether learning was inferior in the second 50 trials (Nemeth et al., 2013).

To determine the amount of declarative knowledge of sequence A, we counted the number of participants who chose the correct sequence in the recognition task I. To compute the recognition score in the recognition task II, we count how many times (normalized by 4) that participants chose the sequence segments that belong to sequence A. To calculate the recall score, we counted the number of correct 2-, 3-, and 4-element chunks in the sequence participants wrote down. These numbers were normalized by the total correct 2-, 3-, and 4-element chunks in sequence A. The recognition score in the recognition task II and recall score were computed for each individual.

### Statistical Analysis

We employed a mixed-effect ANOVA to examine the effects of learning block and age on BMRT/MT. To compare the amount of RT/MT improvements driven by the online and oﬄine processes and their age-related differences, mixed-effect ANOVAs were used. A two-way mixed-effect ANOVA was employed to examine the effects of age and phase of block (i.e., the first and last 50 trials) on sequence learning measured by the first and last 50 trials between blocks 4 and 5. For all mixed-effect ANOVAs, the co-variance matrix was determined by the Akaike’s Information Criterion (AIC). Non-parametric ANOVAs (i.e., Kruskal–Wallis test) were used to examine the age effect on recognition and recall scores. For all ANOVA tests, *post hoc* tests employed a Tukey–Kramer correction. All effects were tested at a significance level *p* = 0.05.

## Results

### Mean RT

**FIGURE 2:**
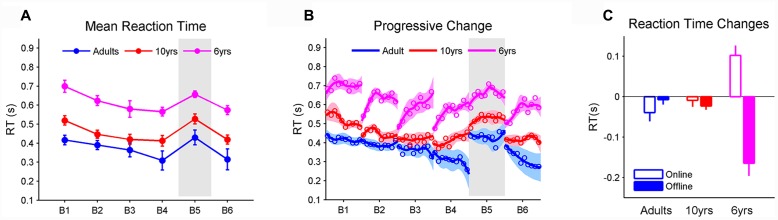
**(A)** The mean reaction time for each block (BMRT). The gray area represents the block in which the stimuli follows a novel sequence. The BMRT depended on learning blocks and age. Adults and 10-year-olds were faster than the 6-year-olds, while there was a trend that 10-year-olds had the same BMRT as adults. All three groups learned sequence A, as revealed by decreased BMRT from blocks 1 to 4 and increased BMRT from blocks 4 to 5. Such learning did not result from motor improvements as the BMRTs in blocks 1 and 5 were the same; **(B)** changes in RT within and between blocks. The solid line represents the trend as RT progressively changes, estimated by a local weighted regression. Shaded areas are the standard errors of the trend. Circles represent RMRTs (i.e., the mean RT of one repetition of stimulus sequence). The adults’ RTs progressively decreased within each block, while there were oﬄine boosts in RT in 6-year-olds; and **(C)** average online and oﬄine RT changes when learning sequence A from blocks 1 to 4. Note that negative values imply decreases in RT (i.e., RT becomes faster). Both oﬄine and online changes in RT relied on age. Specifically, the oﬄine change was greater in 6-year-olds (greater than 0) than 10-year-olds and adults. The online change was greater in 10-year-olds and adults than 6-year-olds whose RT deteriorated ‘online.’ Error bars represent standard errors of the mean performance within each block. RT, reaction time.

Performance was measured by the mean RT of 100 steps in each block (BMRT) and the magnitude of learning was marked by the BMRT difference between sequence A in block 4 and the novel sequence B in block 5 (see Materials and Methods). We found significant effects for learning blocks (*F*_5,29_ = 16.01, *p* < 0.001, η^2^ = 0.41) and age (*F*_2,29_ = 22.18, *p* < 0.001, η^2^ = 0.03) but no interaction on the BMRT (**Figure 2A**). *Post hoc* analyses with a Tukey–Kramer correction found that 10-year-olds appeared to have a longer BMRT than adults (*p* = 0.062). Both groups were faster than the 6-year-olds (*p* < 0.001), whereas all three groups decreased their BMRT (i.e., faster RT) from blocks 1 to 4 (*p* < 0.001). In addition, the BMRT became longer from blocks 4 to 5 where sequence B was introduced (*p* < 0.001). No differences were found between blocks 1 and 5, indicating that the improvement in RT from blocks 1 to 4 was due to sequence learning, and not motor improvements. These results together indicate that all groups learned sequence A to a comparable level. In addition, the same BMRT before (block 4) and after (block 6) sequence B suggests that all age groups preserved the learning of sequence A even after practicing sequence B.

### Online and Oﬄine RT Changes

Visually, it is clear that the three groups demonstrated different RT patterns (**Figure 2B**). The adults’ RTs gradually decreased within each block, while there were oﬄine boosts in RT in 6-year-olds. Ten-year-olds’ RTs exhibited a mixed pattern similar, in part, to the adults and the 6-year-olds.

**Figure 2C** displayed the average amount of oﬄine (between blocks) and online (within blocks) changes that took place when participants learned sequence A through blocks 1 to 4 (see Materials and Methods). It was found that the type of RT improvement (online vs. oﬄine) was significantly interacted with age (*F*_2,29_ = 19.59, *p* < 0.001, η^2^ = 0.52). Tukey–Kramer corrected *post hoc* tests revealed that online and oﬄine RT changes were comparable in adults and 10-year-olds while RT in 6-year-olds improved more during the oﬄine than the online period (*p* < 0.001). In addition, 6-year-olds produced greater oﬄine RT changes (greater than 0; *p* < 0.001, Cohen’s *D* = 1.67) than 10-year-olds (*p* < 0.001) and adults (*p* < 0.001). In contrast, 10-year-olds (*p* < 0.01) and adults (*p* < 0.01) exhibited greater online RT changes than 6-year-olds whose RT deteriorated ‘online’ (less than 0; *p* < 0.001, Cohen’s *D* = 1.34).

### Additional Testing for Fatigue Effect

**FIGURE 3:**
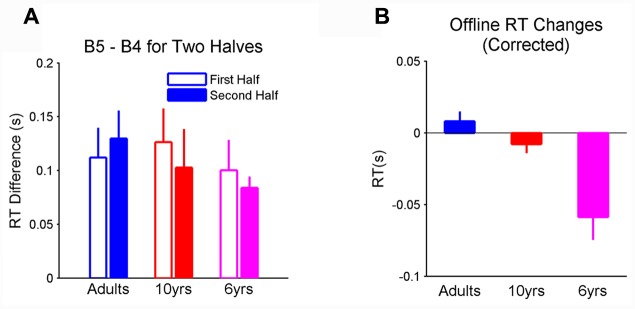
**(A)** Learning measured in both the first and last 50 trials in blocks. Learning magnitudes were comparable between two halves across all three groups; **(B)** corrected oﬄine learning after removing the RT decrement within the preceding block from the total oﬄine gain depended on age. Importantly, the corrected oﬄine RT change was significantly different from 0 in 6-year-olds. Error bars represent standard errors. RT, reaction time.

First, we compared the amount of learning measured by changes in mean RT of the first 50 trials from blocks 4 to 5 and the mean RT of the last 50 trials from blocks 4 to 5. It was found that sequence learning did not depend on age, block phase, and their interaction (**Figure 3A**). These results show that the magnitude of learning measured in the last 50 trials of the blocks was comparable to that in the first 50 trials of the blocks in all groups. Most importantly, 6-year-olds, showed the same magnitude of learning as 10-year-olds and adults in both block phases.

We also computed corrected oﬄine process by removing the RT decrement within the preceding block from the total oﬄine gain. The correction on oﬄine process did not change the age effect (*F*_2,29_ = 12.45, *p* < 0.001, η^2^ = 0.43; **Figure 3B**). Specifically, the corrected oﬄine process was significantly affected by age. The oﬄine RT improvement was significantly greater than 0 (after corrected; *p* < 0.001, η^2^ = 0.1.17) in 6-year-olds and this improvement was greater than that in 10-year-olds (*p* < 0.01) and adults (*p* < 0.001). Taken together, these results suggest that fatigue could not fully explain the oﬄine improvements in RT and its age-related profile.

### Mean MT

**FIGURE 4:**
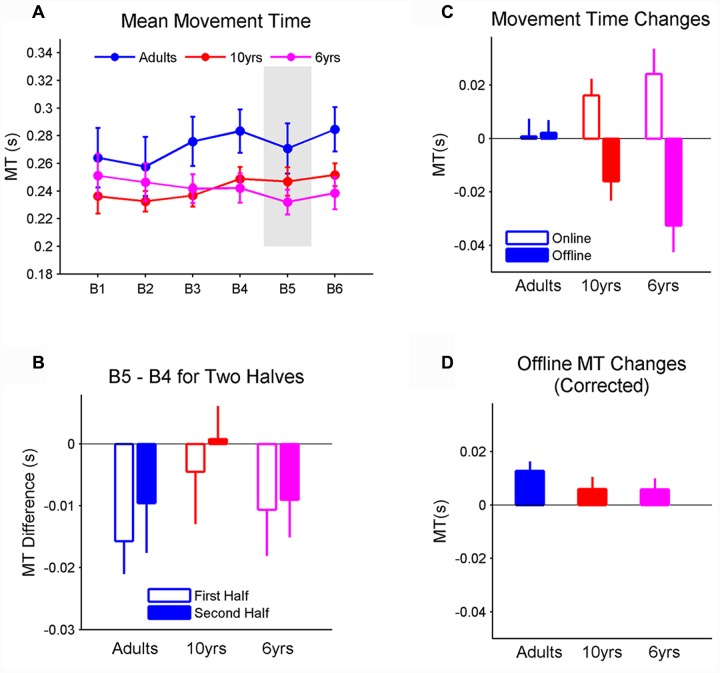
**(A)** The mean movement time (MT) for each block. The gray area represents the block in which the stimuli follows a novel sequence. All three groups had comparable MT. In addition, MT did not change from blocks 4 to 5 in all three groups, suggesting that MT does not represent sequence learning. **(B)** Changes from blocks 4 to 5, in terms of MT, measured in both the first and second halves. The changes were comparable between two halves across all three groups; changes in RT within and between blocks. **(C)** Average online and oﬄine MT changes when learning sequence A from blocks 1 to 4. Note that negative values imply decreases in MT (i.e., MT becomes faster). Online changes in MT did not rely on age. In contrast, oﬄine MT changes significantly depend on age. Specifically, the oﬄine change was greater in 6-year-olds than adults; and **(D)** when the oﬄine gain was corrected by removing the MT decrement within the preceding block from the total oﬄine change, the age effect vanished. Importantly, the corrected oﬄine MT change was no longer significantly different from 0 in 6-year-olds. Error bars represent standard errors of the mean performance within each block. MT, movement time.

Unlike RT, mean MT did not differ between groups. There was a significant effect of block on MT (*F*_2,29_ = 2.95, *p* < 0.05, η^2^ = 0.07) but the MT differences between blocks were within 0.01 s. *Post hoc* tests failed to identify further differences between each pair of blocks (**Figure 4A**). In particular, MT was comparable between blocks 4 and 5, suggesting that MT did not represent sequence learning (Du and Clark, 2016). We further measured the MT changes from blocks 4 to 5 for the first 50 trials and last 50 trials, respectively, and did not find significant effects of age, block phase, and their interaction (**Figure 4B**). When the amounts of online and oﬄine MT changes and their age-related effect were examined, the significant interaction between the type of MT improvement and age was found (*F*_2,29_ = 3.65, *p* < 0.05, η^2^ = 0.17; **Figure 4C**). In particular, online changes in MT were comparable between groups, while oﬄine changes in MT were greater than 0 (*p* < 0.001, η^2^ = 1.03) in 6-year-olds and greater than oﬄine MT changes in adults (*p* < 0.05). However, the significant effect of age vanished when oﬄine MT changes were corrected by removing the MT decrement within the preceding block from the total oﬄine gain. Importantly, the corrected oﬄine MT change in 6-year-olds was no longer significantly different from 0 (**Figure 4D**).

### Declarative Knowledge of Sequence A

**FIGURE 5:**
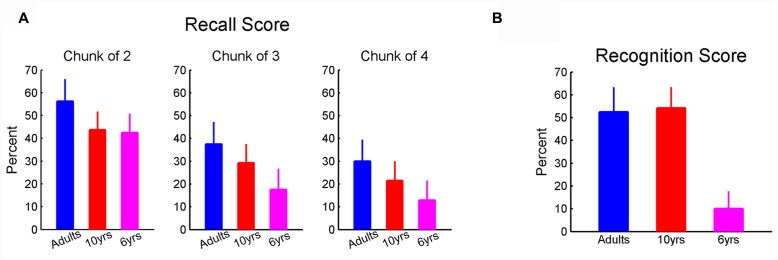
**(A)** The recall score. There was a tendency that the recall score reduced with age decreasing; **(B)** recognition score. Six-year-olds showed lower recognition score compared to adults and 10-year-olds. Error bars represent standard errors.

Upon completion of the six learning blocks, participants completed two recognition tasks and one recall task (see Materials and Methods). The non-parametric ANOVA failed to find a significant effect of age on recall scores for all chunk lengths. Nevertheless, there appears a clear downward trend in recall scores with decreasing age (**Figure 5A**). The failure to observe a statistically significant effect may result from a large dispersion of recall performance in 6-year-olds. Unlike the recall test, we found a significant effect of age on scores (χ_df=2_^2^ = 11.6, *p* < 0.01, η^2^ = 0.37) in the recognition task II where participants were asked to recognized chunks of sequence. Six-year-olds (**Figure 5B**) scored lower than the 10-year-olds and adults. In addition, in the recognition task I where sequence A was given along with the other three irrelevant sequences, eight adults (out of 10), nine 10-year-olds (out of 12), and none of the 6-year-olds were able to identify the correct sequence.

## Discussion

Our results demonstrate that both adults and children learn motor sequences quickly within one learning session. However, learning in 6-year-olds exhibited oﬄine enhancements in RT, while learning in adults was expressed by online improvements in RT. Ten-year-olds’ RTs exhibited both online and oﬄine changes. In addition, 6-year-olds acquired less declarative knowledge of sequence A compared to 10-year-olds and adults.

Our results reflect similar findings to previous literature on SRT experiments in that both children and adults learn the sequences at a similar rate (Meulemans et al., 1998; Karatekin et al., 2007; Weiermann and Meier, 2012). Given that our task involved moving the legs and maintaining postural control of the whole body rather than simply controlling four fingers, this finding reinforces the veracity of the previous literature findings. What is new here is the differentiation of two behavioral expressions of sequence learning and their age-related use. Despite learning at the same rate, 6-year-olds and adults have very different behavioral expressions of sequence learning. In adults, it has been reported that learning a motor sequence normally starts with an initial stage known as fast learning (Doyon and Benali, 2005). With a short period of practice (i.e., a single learning session) of a new sequence, fast learning produces considerable improvements in the performance (Nissen and Bullemer, 1987; Honda et al., 1998). This initial stage of sequence learning in adults is known to be driven by a trial-by-trial online learning (Cleeremans and McClelland, 1991; Bornstein and Daw, 2012, 2013; Verstynen et al., 2012) as well as an oﬄine process that develops after a short break without physically performing the task (Du et al., 2016). However, unlike adults, we observed that learning in 6-year-olds was reflected only in the oﬄine process. Specifically, 6-year-olds’ RT worsens within blocks and improves oﬄine between blocks. The subsequent question becomes, what are the factors or mechanisms underlying these age-related differences in online and oﬄine processes?

One factor that may result in the age-related differences in behavioral patterns involves the experimental set-up of the task pacing condition. In our study, children and adults performed the SRT task under the same ISI of 1500 ms. Because the adults performed the task quicker than the children (reflected in the RT), the response time, the summation of RT and MT (MT was comparable between groups), was clearly longer in children (e.g., around 1200 ms in block 1) than adults (e.g., around 800 ms in block 1), which yielded a relatively shorter response-stimulus-interval in children (e.g., around 300 ms in block 1). Therefore on the one hand, the 6-year-olds may not be able to learn the sequence online as there was not an adequate amount of time before the next stimulus appears for the required iterative mental computations. On the other hand, the response-stimulus-interval may be long enough for the iterative mental computations and thus the 6-year-olds could have learned the sequence online. However, the online process may not be behaviorally expressed as the iterative mental computation after each step further shortened the amount of time available before the next stimulus appears and thus may interfere with the preparation of the succeeding step, which subsequently prolongs the following RT. To further test the task pacing effect, in a subsequent study from our lab, adults and children performed a self-paced SRT task where the ISI was determined by an individual’s own response speed (Du, 2016). That is, the response-stimulus-interval was fixed at 700 ms for both children and adults and the children’s slower response speeds yielded longer ISI. Such self-determined ISI eliminated the confounding effect of task pacing between children and adults. In this subsequent study, we observed greater oﬄine RT improvements in children compared to adults while learning in adults was primarily accompanied by an online process. Furthermore, if the online process is affected by task pacing, then one would expect less online RT increases in the subsequent study as the response-stimulus-interval was longer than that in the current study (i.e., 700 ms vs. about 300 ms). However, the magnitudes of online RT increases were very similar (i.e., about 100 ms at the age of six) between the current and subsequent study. Although future studies are necessary to systematically examine the effect of task pacing on the learning process in children (i.e., under a variety of ISI and response-stimulus-interval), the results provide preliminary evidence that the age-related online and oﬄine processes observed in the present experiment may not be a by-product of the task pacing condition.

A second, related, factor that may underlie the online RT deterioration and oﬄine RT improvement is the emergence of fatigue (Ammons, 1947; Denny et al., 1955; Bourne and Archer, 1956; Rickard et al., 2008; Brawn et al., 2010; Rieth et al., 2010). That is, fatigue accumulates and worsens the performance when an individual is practicing the task. This effect dissipates after a rest, leading to an artificial oﬄine enhancement effect. The fatigue explanation is more critical in developmental studies as fatigue is more likely to accumulate in children than adults when they perform the same task. In fact, in our task, a shorter post-response time in children ensured that each new trial come round relatively faster with the potential to produce even greater fatigue compared to adults. This in turn could lead to illusory oﬄine improvements.

However, several observations suggest that the fatigue effect could not fully explain the age-related online and oﬄine processes. First, our data show that RT increased as soon as children started to perform the task (i.e., the first 50 steps in the first block; **Figure 2B**). It is not very likely that fatigue caused the worsened RT at the very beginning of the first block. Additionally, consistent with previous studies (Meulemans et al., 1998; Thomas and Nelson, 2001; Weiermann and Meier, 2012), we observed that, like adults, 6-year-olds successfully learned the sequence. If fatigue appeared as soon as children started to perform the task, it is unlikely that their learning would rise quickly and to a comparable level as the adults who did not exhibit fatigue. Second, sequence learning may depend on the phase of blocks if it is under the influence of fatigue (Nemeth et al., 2013). However, we observed comparable magnitudes of learning as measured by the last 50 trials and the first 50 trials. In addition, regardless of the block phase, learning was comparable between 6-year-olds and the other two groups. This observation also contradicts the hypothesis that fatigue could have a detrimental effect on motor learning (Ammons, 1947; Denny et al., 1955; Bourne and Archer, 1956). Last but not least, if the oﬄine process is an artifact of fatigue, oﬄine improvements must be caused by the performance deterioration that takes place before the rest. MT data support this hypothesis since we observed greater oﬄine improvements in MT in 6-year-olds. This superiority, however, disappeared after the oﬄine MT improvement was corrected and this corrected MT in 6-year-olds did not improve oﬄine. In contrast, oﬄine RT improvements, whether corrected or not by removing the RT decrement within the preceding block, displayed the same age-related differences. In addition, 6-year-olds had significant corrected oﬄine RT improvements, suggesting that fatigue is not the only resource underlying the oﬄine improvement in RT. Rather, the fatigue effect may be combined with some active learning mechanisms, both of which lead to the oﬄine process (Eysenck, 1965).

Finally, the age-related differences in online and oﬄine processes may arise from certain underlying learning mechanisms. For example, the online and oﬄine processes may depend on the degree of task implicitness. The SRT task has been considered an implicit sequence learning task given that participants are not given any information about the sequence throughout the task. However, as our post-test questionnaire revealed, declarative knowledge of the behaviorally learned sequence developed as adults performed the task as indicated by their verbal recall/recognition of, at least, a part of the sequence. These results are consistent with the literature (Cleeremans et al., 1998; Destrebecqz and Cleeremans, 2001). Importantly, we observed that this blended implicit and explicit learning in adults was primarily reflected through the online process. In another experiment from our lab, we found that when the SRT task was made more implicit by asking adults to learn a complex probabilistic sequence, adults did not acquire any declarative sequence knowledge and the learning process was oﬄine rather than online (Du et al., 2016). This is perhaps because explicit knowledge inhibited implicit learning (Destrebecqz and Peigneux, 2005) and thus inhibited the oﬄine (without sleep) RT improvement (Brown and Robertson, 2007a,b) – a salient feature of implicit learning (Robertson et al., 2004). Consistent with this evidence, in the present study there is a parallel between the acquisition of less declarative knowledge, as evidenced by their poor performance in the tasks of recalling and recognizing the sequence, and the more notable oﬄine process in 6-year-olds. This raises the possibility that the age-related online and oﬄine processes may, at least in part, be associated with the implicitness of the sequence learning task. Along this line of thought, one might expect that the learning in 6-year-old children would exhibit online improvements if they learned a short sequence (i.e., shorter than a 10-item sequence) during which declarative knowledge of the sequence might develop. On the other hand, the oﬄine RT enhancement in 6-year-old children may reflect a learning strategy instead of an explicit/implicit learning-relevant process. Unlike trial-by-trial online processing where learning is stochastic and incremental (Cleeremans and McClelland, 1991; Bornstein and Daw, 2012, 2013; Verstynen et al., 2012), oﬄine processing may not update the sequence information until the whole sequence is completed (i.e., after each block). However, this batch learning requires a larger memory capacity to store all sequence elements before they can be learned all at once. This seems to contradict the common fact of limited memory capacity in children compared to adults. Clearly, underpinning mechanisms of the age-related online and oﬄine processes require future investigation.

In summary, we found that learning a foot stepping sequence provides comparable results to finger tapping sequences in which 6-year-olds learn at the same rate as adults. In addition, we found that both online and oﬄine improvements in RT were present and age-related. Learning in the adults is expressed by online enhancements in RT, whereas the 6-year-olds show only oﬄine RT improvements. Learning in 10-year-olds is reflected by both online and oﬄine improvements in RT. These age-related processes may partially originate from a fatigue effect, but it is also likely that they reflect underlying learning strategies. This speculation needs to be further elucidated in future studies.

## Ethics Statement

This study was approved by the Institutional Review Board at the University of Maryland, College Park and it was performed in accordance with the approved guidelines. Signed written informed consent forms from the adult participants/parents and assent forms from child participants were received prior to their participation.

## Author Contributions

YD, NV, MK, JW, and JC designed the experiment. YD, NV, and MK collected the data. YD analyzed the data and prepared all figures. YD drafted the manuscript. JW and JC provided critical revisions. All authors approved the manuscript to be published.

## Conflict of Interest Statement

The authors declare that the research was conducted in the absence of any commercial or financial relationships that could be construed as a potential conflict of interest.
